# Dysregulation of Inflammatory Pathways in Adult Spinal Deformity Patients with Frailty

**DOI:** 10.3390/jcm13082294

**Published:** 2024-04-16

**Authors:** Tomohisa Tabata, Mitsuru Yagi, Satoshi Suzuki, Yohei Takahashi, Masahiro Ozaki, Osahiko Tsuji, Narihito Nagoshi, Morio Matsumoto, Masaya Nakamura, Kota Watanabe

**Affiliations:** 1 Department of Orthopedic Surgery, Keio University School of Medicine, Tokyo 160-8582, Japan; 2 Department of Orthopedic Surgery, School of Medicine, International University of Health and Welfare, Otawara 324-8501, Japan; 3 International University of Health and Welfare Narita Hospital, Narita 286-8520, Japan

**Keywords:** frailty, adult spinal deformity, biomarker, complication, HRQOL

## Abstract

**Background/Objectives**: An important aspect of the pathophysiology of frailty seems to be the dysregulation of inflammatory pathways and the coagulation system. However, an objective assessment of the impact of frailty on the recovery from surgery is not fully studied. This study sought to assess how frailty affects the recovery of adult spinal deformity (ASD) surgery using blood biomarkers. **Methods**: 153 consecutive ASD patients (age 64 ± 10 yr, 93% female) who had corrective spine surgery in a single institution and reached 2y f/u were included. The subjects were stratified by frailty using the modified frailty index-11 (robust [R] group or prefrail and frail [F] group). Results of commonly employed laboratory tests at baseline, 1, 3, 7, and 14 post-operative days (POD) were compared. Further comparison was performed in propensity-score matched-39 paired patients between the groups by age, curve type, and baseline alignment. A correlation between HRQOLs, major complications, and biomarkers was performed. **Results**: Among the propensity-score matched groups, CRP was significantly elevated in the F group at POD1,3(POD1; 5.3 ± 3.1 vs. 7.9 ± 4.7 *p* = 0.02, POD3; 6.6 ± 4.6 vs. 8.9 ± 5.2 *p* = 0.02). Transaminase was also elevated in the F group at POD3(ASD: 36 ± 15 vs. 51 ± 58 U/L, *p* = 0.03, ALT: 32 ± 16 vs. 47 ± 55 U/L, *p* = 0.04). Interestingly, moderate correlation was observed between transaminase at POD1 and 2 y SRS22 (AST; function r = −0.37, mental health r = −0.39, satisfaction −0.28, total r = −0.40, ALT; function r = −0.37, satisfaction −0.34, total r = −0.39). **Conclusions**: Frailty affected the serum CRP and transaminase differently following ASD surgery. Transaminase at early POD was correlated with 2 y HRQOLs. These findings support the hypothesis that there is a specific physiological basis to the frailty that is characterized in part by increased inflammation and that these physiological differences persist.

## 1. Introduction

Surgery for spinal disease sometimes requires longer surgical time and higher blood loss. Especially corrective spine surgery for adult spinal deformity (ASD) is often required for long surgical time, large dissection, and higher blood loss, which results in a relatively higher complication rate and revision rate [1,2,3,4]. Due to the nature of the surgery, assessing the risk of complications and poor recovery from surgery is essential to optimize the outcome and minimize the complications in ASD surgery [5,6]. Several risk factors have been reported for surgical complications and inferior clinical outcomes, including older age, history of diabetes mellitus, smoking, depressive mental status, and frailty [4,5,6,7,8]. Among them, frailty is gaining attention as an important risk for the development of surgical complications and inferior clinical outcomes in various types of surgeries and diseases [6,7,8,9,10].

Several recent studies have described the role of frailty in the development of postoperative medical and surgical complications as well as recovery from surgical invasion in spine surgery [6,7,8,9,10]. Parfentjev first described frailty as a clinically recognizable state of increased vulnerability resulting from an aging-associated decline in reserves and function across multiple systems, such that the ability to cope with everyday or acute stressors is compromised [11]. Yagi et al. described that surgical outcomes and complication rates deteriorated as frailty advanced in ASD patients [7].

The diagnosis of frailty is usually made based on clinical criteria, mostly using the individual’s medical history and activity [9,12,13,14]. Recently, several assessment tools were reported, including the Canadian Study of Health and Aging Frailty Index (CHSA-FI) and the 11-item modified frailty index (MFI-11) [12,13,14]. Although the usefulness of mFI-11 has been described, it can be difficult to comprehensively assess a patient’s frailty because past medical history does not represent the present state of each illness. Therefore, there is an increasing need to identify robust biomarkers for frailty.

Several recent studies described that the important aspect of the pathophysiology of frailty might be the dysregulation of inflammatory pathways and of the coagulation system [11,15,16,17,18,19,20,21,22,23,24,25]. Saedi et al. described that decreased serum hemoglobin, increased albumin, and C-related protein (CRP) are the biomarkers that can distinguish the presence of frailty from the commonly employed laboratory tests [26]. Li et al. described that there is a heightened inflammatory state in other intermediary pathophysiologic processes in the pathogenesis of frailty [15]. However, these findings are not consistently described in the other literature.

Acknowledging the pressing need for dependable biomarkers of frailty, our study endeavors to determine how frailty influences postoperative recovery in ASD surgery by examining a spectrum of blood biomarkers. This investigation aims to connect biomarker fluctuations with clinical outcomes, thereby enhancing our understanding of frailty in a surgical context.

The biomarkers analyzed in this study include the following, which were measured at baseline and on postoperative days (POD) 1, 3, 7, and 14: white blood cell (WBC), red blood cell (RBC), hemoglobin (Hb), hematocrit (HCT), platelet count (PLT), total leucocyte count (TLC), total protein (TP), albumin (ALB), blood urea nitrogen (BUN), creatinine (CRE), aspartate aminotransferase (AST), alanine aminotransferase (ALT), AST to ALT ratio (AAR), activated partial thromboplastin time (aPTT), prothrombin time (PT), and international normalized ratio (INR). These biomarkers were selected for their potential to provide insight into the physiological impact of ASD surgery and the concurrent state of frailty.

## 2. Materials and Methods

### 2.1. Patient Population

Ethical compliance and patient consent are the bedrocks of our research. Adhering to the Helsinki Declaration, our study received approval from the institutional review board. All participating subjects provided informed consent, affirming their understanding and agreement to partake in the study. The methodologies we employed were consistent with established guidelines and regulations. We retrospectively reviewed charts and radiographs for 169 consecutive patients who underwent surgery for ASD in the academic hospital between 2012 and 2018.

### 2.2. Inclusion and Exclusion Criteria

Subjects were at least 40 years old at the index surgery and had a spinal deformity defined by a Cobb angle ≥ 20°, a C7 sagittal vertical axis (C7SVA) ≥ 5 cm, or pelvic tilt (PT) ≥ 25° [27]. We included patients with at least 5 fused vertebrae, segmental instrumentation and fusion from the upper-instrumented vertebra (UIV) to the lower-instrumented vertebra (LIV), and complete 2-year follow-up data. Patients were excluded if they had complications such as surgical site infection (SSI), urinary tract infection (UTI), or revision surgery during the perioperative period.

### 2.3. Collection of Radiographic, Health-Related Quality of Life (HRQOL), Laboratory Data, and other Demographic Data

We collected demographic and clinical data for each patient, including age, gender, body mass index (BMI), bone mineral density (BMD), smoking status, history of joint arthroplasty (hip), and spine surgery. Frailty and comorbidities were assessed using the 11-item modified frailty index (MFI-11).

We collected the following surgical data: the SRS-Schwab ASD classification, application of three-column osteotomies, lateral lumbar interbody fusion (LLIF), UIV and LIV levels, number of fused vertebrae, estimated blood loss, and time of surgery.

Radiographic data obtained at baseline and 2-year follow-up included the following measurements: Cobb angle, C7SVA, PT, and pelvic incidence (PI).

As a surrogate for HRQOL, we used the Scoliosis Research Society-22r questionnaire (SRS22r) results at baseline and at the 2-year follow-up.

**Figure 1:**
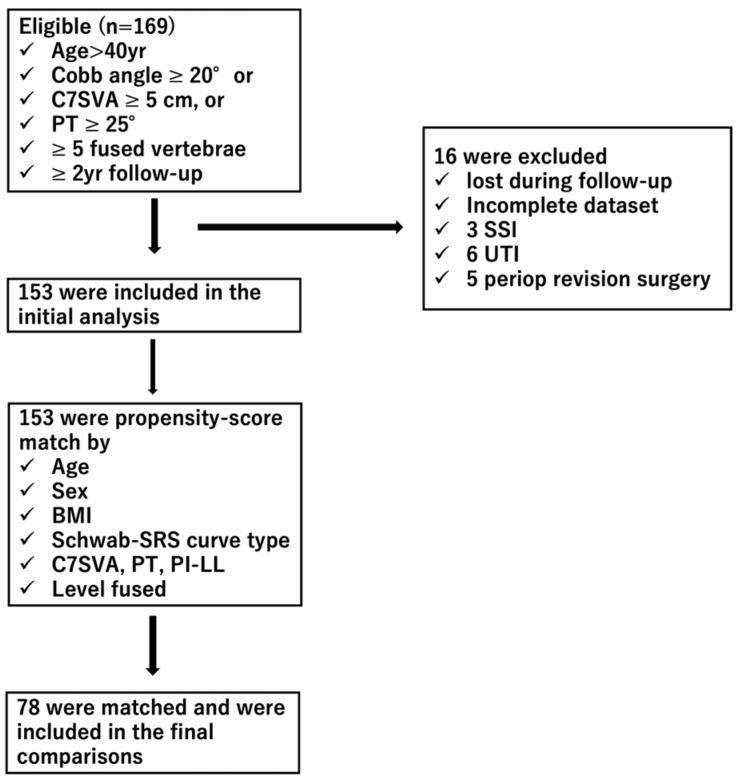
Patient flow diagram.

The commonly employed laboratory test data were obtained at baseline, 1,3,7, and14 days post-operative day (POD) after index surgery (WBC, RBC, Hb, HCT, PLT, TLC, TP, ALB, BUN, CRE, AST, ALT, AST to ALT ratio (AAR), aPTT, prothrombin time, and PT/INR). Of the 169 candidates, 153 subjects had complete demographic, radiographic, and laboratory data that sufficiently captured the role of frailty in the inflammatory reaction following surgery and were thus included in the study cohort (Figure 1).

We conducted a retrospective review of 169 ASD patients who underwent corrective spine surgery at our institution. Of these, 153 patients were included. After propensity score matching, 78 patients were compared.

### 2.4. Propensity Score Matching of the Patient Cohort (Figure 1)

To mitigate confounding factors and potential biases, we employed propensity score matching. Matching variables included age, sex, BMI, the SRS-Schwab ASD classification, and baseline spinal alignment, among others. This created a balanced comparison between the robust and prefrail/frail groups. Propensity scores were calculated by a linear regression analysis of these parameters. The chi-square value of the Hosmer-Lemeshow goodness of fit statistic for this propensity score matching was 5.20, and the *p*-value was 0.737, indicating good model adaptation.

### 2.5. Statistical Analysis

Patients were stratified into robust, prefrail, and frail categories according to the mFI-11 (robust: mFI-11 = 0, prefrail: mFI-11 < 0.27, frail: mFI-11 ≥ 0.27) [12,13]. Additionally, the patients were grouped into a Robust (R) group or a Prefrail/frail (F) group. Relationships between frailty, HRQOLs, complications, and baseline and post-operative laboratory data were analyzed. Statistical analyses were then performed to ascertain the associations between frailty and various outcomes. Univariate analyses were conducted, and variables were tested for normality [28]. Mean ± SD values were reported. We applied various statistical tests, including the unpaired *t*-test, chi-square test, Tukey’s HSD test, and Fisher’s exact test, where appropriate. A *p*-value of less than 0.05 was indicative of statistical significance, with all calculations performed using SPSS version 27.0 (Chicago, IL, USA).

## 3. Results

### 3.1. Characteristics of the Patient Cohort

The patient characteristics are shown in Table 1. Of the 153 patients, 47 (31%) were categorized as prefrail, and 9 (6%) were categorized as frail, while the remaining 97 (63%) were categorized as robust by the mFI-11.

There were significant differences in baseline values of age, BMD, and curve type between the Robust group and Prefrail/frail groups, while similar values were observed for gender, BMI, the level used, the level of LIV application of LLIF, PSO, and the prevalence of revision surgery.

Additionally, time of surgery and major complication rate were both worse in the Prefrail/frail patient group (Table 1). Comparisons of baseline spinal alignment indicated more severe malalignment in the Prefrail/frail patient group when compared with those in the Robust patient group (Table 2). Similarly, a trend toward inferior baseline SRS22 function and pain domain score was seen in the Prefrail/frail patient group, though not statistically significant (Table 2). The sagittal alignment was worse in the Prefrail/frail patient group at 2 years post-operatively (Table 3). Similarly, a trend toward inferior SRS22 function, pain, and the total score was observed in the Prefrail/frail patient group at 2 years postoperatively, though not significant (Table 3).

### 3.2. Comparisons of Baseline and Post-Operative Laboratory Data between the Robust Group and Prefrail/Frail Group

**Figure 2:**
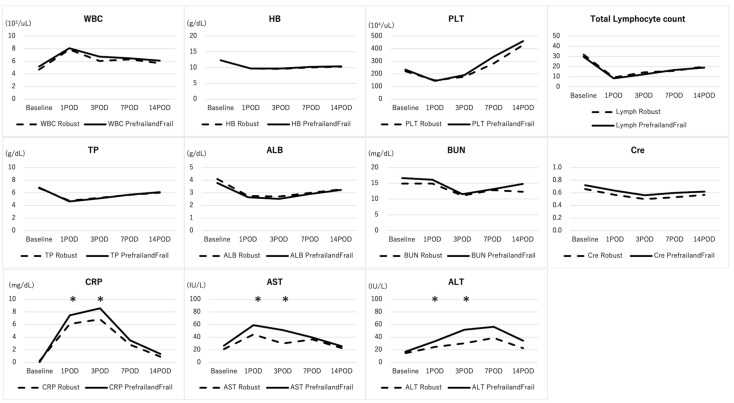
Comparison of blood biomarkers between robust and prefrail/frail patients following ASD surgery. The baseline laboratory data was similar between the R group and the F group. Postoperatively, CRP was significantly elevated in the F group when compared with the R group at POD1,3. Transaminase was also elevated in the F group at POD1,3. * indicates statistically significant.

The baseline laboratory data were similar between the Robust group and the Prefrail/frail group (Figure 2). Regarding the coagulation pathways, aPTT and PT-INR were both similar between the 2 groups (Figure 2). None of the patients had abnormal baseline aPTT or PT-INR.

Postoperatively, CRP was significantly elevated in the Prefrail/frail group when compared with the Robust group at POD1,3 (R vs. F group: POD1; 6.1 ± 3.3 vs. 7.5 ± 4.2, *p* = 0.03, POD3; 6.8 ± 4.2 vs. 8.6 ± 4.8, *p* = 0.02). Transaminase was also elevated in the Prefrail/frail group at POD1, 3 (R vs. F group: POD1 AST; 47.6 ± 23.6 vs. 0.76.0 ± 168.4 U/L, ALT; 24.7 ± 11.2 vs. 41.0 ± 91.5 U/L, *p* = 0.04, 0.02, POD3 AST; 34.9 ± 15.1 vs. 0.47.2 ± 54.9 U/L, ALT; 31.7 ± 16.4 vs. 0.42.9 ± 61.6 U/L, *p* = 0.04, 0.04), while no difference was observed in other factors (Figure 2).

### 3.3. Comparisons of Baseline and Post-Operative Laboratory Data in the Propensity-Score Matched Patient Cohort

**Figure 3:**
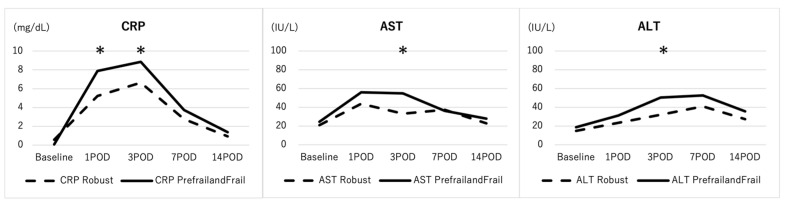
Comparison of serum CRP and transaminase between robust and prefrail/frail patients following ASD surgery in a propensity-score-matched cohort. CRP was significantly elevated in the F group when compared with the R group at POD1,3 and transaminase was also elevated in the F group at POD3. * indicates statistically significant.

Similarly, aPTT and PT-INR were similar between the 2 groups (Figure 3). Seventy-eight patients were matched for propensity score, resulting in patients in the R group and F group (Table 4). In addition, gender, the Schwab-SRS curve type, application of pedicle-subtraction osteotomy (PSO), application of lateral lumbar interbody fusion (LLIF), and LIV level (pelvis) were similar between the Robust patient and Prefrail/frail patient groups (Table 4). Among the 78 propensity-score-matched patients, CRP was significantly elevated in the Prefrail/frail group at POD1,3, and transaminase was also elevated the in Prefrail/frail group at POD3 (Figure 3).

### 3.4. Correlation between Frailty (mFI-11) and Baseline Biomarkers in the Prefrail and Frail Patient Group

There were moderate correlations between the mFI-11 and baseline PLT count and between the MFI-11 and PT-INR (PLT; r = −0.294, PT-INR; r = 0.354), while no correlation was found between the mFI-11 and other baseline biomarkers.

### 3.5. Correlation between Baseline Biomarkers and Baseline and 2-Year Post-Operative HRQOLs in the Prefrail and Frail Patient Group

Correlation coefficient analyses indicated that the baseline serum CRP level was moderately correlated with the baseline SRS22 pain score in the prefrail/frail group, while none of them had inflammatory disorders or collagen diseases (r = −0.508). Interestingly, a moderate correlation was found between the baseline PLT count and SRS22 pain, self-image, and total score at 2 years post-operatively (r = −0.312, −0.327, and −0.288, respectively).

### 3.6. Correlation between Post-Operative Serum CRP Level, Transaminase, and 2-Year Post-Operative HRQOLs in the Prefrail and Frail Patient Group

There was no correlation between serum CRP level and 2-year post-operative SRS22 scores. However, moderate correlations were observed between transaminases at POD1 and 2-year post-operative SRS22 outcomes (AST; function r = −0.373, mental health r = −0.386, satisfaction −0.283, total r = −0.399, ALT; function r = −0.367, satisfaction −0.336, total r = −0.389).

Correlation analyses between serum CRP and transaminase showed a moderate correlation between POD1 serum CRP level and both AST and ALT at POD3 and POD7 (POD3; AST: r = 0.61 *p* < 0.01, ALT: r = 0.61 *p* < 0.01, POD7; AST: r = 0.43 *p* < 0.01, ALT: r = 0.43 *p* < 0.01), but no correlation was found in POD14. The same trend was found between POD3 serum CRP and AST and ALT at POD3,7 (POD3; AST: r = 0.45 *p* < 0.01, ALT: r = 0.44 *p* < 0.01, POD7; AST: r = 0.32 *p* < 0.01, ALT: r = 0.39 *p* < 0.01).

### 3.7. Comparison of Baseline Biomarkers between ASD Patients Who Developed Severe Adverse Events during Follow-Up and Those Who Did Not in Prefrail/Frail Patients

Among the Prefrail/frail group, a significant difference was observed for baseline serum creatinine concentration between ASD patients who developed SAE and those who did not (SAE+ vs. SAE−; serum creatinine: 0.78 ± 0.23 vs. 0.67 ± 0.18 mg/dL, *p* = 0.05, Table 5). Similar findings were observed when comparing the baseline biomarkers between ASD patients who developed severe adverse events during follow-up and those who did not in the entire population (Supplementary Table S1).

## 4. Discussion

### 4.1. Baseline Difference for Biomarkers between Robust and Prefrail/Frail Patients in ASD Surgery

In the present study, no significant difference was observed for baseline biomarkers between robust and prefrail/frail patients in ASD surgery, including inflammatory pathways and the coagulation system. A common feature of frailty is the demonstrable activation of inflammatory and coagulation pathways, and these are currently the target of interventional trials [11,15,16,17,18,19,20,21,22,23,24,25]. Kanapuru B et al. described that frail and prefrail patients from the Cardiovascular Health Study had significantly higher levels of fibrinogen, factor VIII, and D-dimer levels compared with the robust patients [17]. Patel A et al. described that among 4263 patients with cardiac disease, older frail patients had higher baseline IL-6 and CRP and higher postoperative IL-6 levels (<12 h and >1 week) of 0.25. Unlike the aforementioned studies, there were no quantitative differences in baseline biomarkers between robust and prefrail/frail patients in this study. This difference may partly be because of the different nature of the pathology between cardiac disease and ASD.

ASD surgery is typically an elective procedure, and patients undergo thorough preoperative assessments to ensure their readiness for the intervention. In contrast, cardiac disease often necessitates immediate treatment. This distinction in disease urgency and the preoperative evaluation process may partly explain the absence of quantitative differences in baseline biomarkers between robust and prefrail/frail patients in our study. While frailty is characterized by heightened inflammation and coagulation in various contexts, the elective nature of ASD surgery may mitigate some of these baseline differences.

### 4.2. The Elevation of Inflammatory Pathway and Coagulation Pathway Biomarkers in ASD Patients

Previous literature described that both inflammatory and coagulation pathways are increasingly active with advanced age and who meet the criteria for frailty. The coexistence of these factors may suggest the interaction of inflammation and coagulation in the pathophysiology of frailty. Inflammatory cytokines are known to stimulate the release of procoagulant factors in various cell types, and it has been suggested that the release of the increased cytokine in frailty patients may be involved in the activation of coagulation.

We found a significant correlation between the baseline PT count, PI-INR, and severity of frailty. There were moderate correlations between the mFI-11 and baseline PLT count and between the mFI-11 and PT-INR. Moreover, frailty affected the serum CRP level and transaminase level differently in the early postoperative days.

The present study indicated that corrective spine surgery for ASD is typically highly invasive, requiring longer surgical time and greater blood loss. This nature of surgery may stress the liver function, requiring multiple drug metabolisms, ischemia, and anesthesia, which resulted in the elevation of transaminase, especially in frail patients.

### 4.3. The Correlation between Inflammatory Biomarkers and Clinical Outcomes in ASD Patients

In the present study, although the baseline serum CRP level in the prefrail and frail patient cohorts was not different from that in the robust patient cohort, the baseline serum CRP level was moderately correlated with the baseline SRS22 pain score in the prefrail/frail group, since this correlation was not found in the robust patient cohort. Additionally, no correlation was found between serum CRP level at any post-operative time period and 2-year post-operative SRS22 scores. These findings are in line with the literature indicating that sterile inflammatory responses and associated hematological biomarkers are significantly relevant in the postoperative period for elderly patients undergoing surgical procedures [29,30]. It is therefore plausible that, similar to the ratios indicating sterile inflammation in the context of orthopedic surgeries, the transient postoperative elevations of CRP and transaminase levels in our study serve as reflections of an inflammatory response. This response does not directly lead to poor outcomes but underscores a state of vulnerability in frail patients that predisposes them to such risks. Plas M et al. reviewed the biomarkers and complications in 224 elderly patients undergoing surgery for a solid malignant tumor and described that there were associations between the inflammatory response to surgery and postoperative complications, especially in early postoperative IL-1 and IL-6 [25]. In the present study, unfortunately, we did not harvest baseline and post-operative IL-1 and IL-6; therefore, direct comparisons with those in the previous study cannot be undertaken. However, the elevated early post-operative CRP in prefrail/frail patients indicated the activation of an inflammatory response in frail patients following highly invasive surgery such as ASD surgery. Further detailed study warrants the early perioperative inflammatory response to improve perioperative care and might help to mitigate the risk of ASD surgery in frail patients. The sterile inflammatory response, as seen in the context of subtrochanteric fractures treated with different surgical techniques, showcases the importance of understanding how blood-derived biomarkers are indicative of postoperative immune changes, particularly in the elderly [29,30]. Similarly, in our study, the early post-operative elevation of CRP among prefrail/frail patients suggested heightened sterile inflammation following the invasiveness of ASD corrective surgery. While we have not measured the same biomarkers as the study on subtrochanteric fractures, such as the NLR, PLR, MLR, SII, SIRI, and AISI ratios, our findings on CRP levels support the notion that sterile inflammation markers could serve as indicators of postoperative immune response in ASD patients. Future studies may benefit from a comprehensive assessment including these ratios to validate the sterile inflammatory response and optimize perioperative care for ASD surgery, particularly in frail individuals.

### 4.4. The Correlation between Biomarkers and Clinical Outcomes and Severe Adverse Events in ASD Patients

In the present study, a moderate correlation was found between the baseline PLT count and SRS22 pain, self-image, and total score at 2 years post-operatively. Moderate correlations were also observed between transaminase levels at POD1 and 2-year post-operative SRS22 outcomes. Maeda D, et al. reviewed 1327 elderly patients hospitalized with heart failure [20]. They described that a high AST to ALT ratio was identified as an independent marker of frailty and was associated with lower physical performance. Vespasiani-Gentilucci U et al. also described that abnormal ALT levels are correlated with frailty and reduced survival in elderly sublets without chronic liver disease, malignancies, or alcohol abuse [19]. In the present study, elevated post-operative transaminase levels were observed in prefrail/frail patients and were found to be correlated with poor clinical outcomes. This association may be attributed to the fact that liver function is closely interconnected with overall health indicators such as vitality and physical activity. In frail patients, disrupted liver function could contribute to the development of adverse clinical outcomes in ASD surgery.

Collectively, these findings indicate that transient postoperative elevations in serum CRP or AST/ALT do not directly cause poor surgical outcomes. Rather, they suggest that patients with frailty who experience dysregulation of inflammatory pathways and coagulation systems are at a higher risk of unfavorable surgical outcomes. Furthermore, a comparison of baseline biomarkers between ASD patients who developed SAE and those who did not in prefrail/frail patients showed that baseline serum creatinine concentration was associated with the development of SAE. Wilhelm-Leen ER et al. described that frailty is significantly associated with all stages of chronic kidney disease, particularly moderate-to-severe chronic kidney disease [22]. Shlipak et al. evaluated the prevalence of frailty in an elderly cohort and found that 15% of frailty patients had an elevated serum creatinine concentration [31]. The findings of the present study follow the previous literature.

The present study was limited by its retrospective design, which precludes drawing firm conclusions about the biomarker of frailty and its role in the clinical outcomes and development of SAEs following ASD surgery. While this study has provided valuable insights, it is pertinent to highlight that future prospective studies should consider a broader range of biomarkers, as noted in Moldovan et al., to thoroughly assess sterile inflammation postoperatively [29,30]. This will enhance our understanding of the impact of inflammatory responses on clinical outcomes and the development of SAEs following ASD surgery. Additionally, we enrolled patients from academic hospitals in an East Asian country, so the patients were mostly Asian. Therefore, our results cannot necessarily be extrapolated to all other hospital settings. Furthermore, we did not collect data on the amount of non-steroidal anti-inflammatory drugs (NSAIDs) administered postoperatively, which could have influenced inflammatory biomarker levels. These limitations should be considered when interpreting our results.

However, the present study clearly described that there is a specific physiological basis to the frailty that is characterized in part by increased inflammation and elevated markers of blood clotting and that these physiological differences persist. Furthermore, several baseline and early post-operative biomarkers, such as ALT/AST and serum creatinine level, are able to predict the development of SAE as well as inferior clinical outcomes following ASD surgery. Combination use of an individual’s medical history, physical function, and these biomarkers may improve the preoperative assessment of ASD patients requiring surgery.

Further analyses, including different patient populations, will be necessary to validate the biomarker of frailty and its role in the clinical outcomes and development of SAEs following ASD surgery.

## 5. Conclusions

In conclusion, this study has highlighted the association between frailty, specific inflammatory and coagulation biomarkers, and postoperative outcomes in patients undergoing ASD surgery. We found that while frailty correlates with elevated levels of certain biomarkers, these changes do not necessarily predict poorer surgical outcomes directly but rather indicate a heightened state of vulnerability, which may influence recovery. The study underscores the importance of considering frailty and biomarker levels in preoperative assessments to potentially tailor surgical approaches and postoperative care. We recognize limitations such as the retrospective design and the homogeneity of our patient population, which may restrict the generalizability of our findings. Therefore, prospective studies involving a broader demographic will be critical to validate these biomarkers’ roles in managing ASD surgery patients effectively, and to determine whether interventions targeting these biomarkers before surgery could modify outcomes. These findings lay the groundwork for future research to explore how biomarkers can be used to refine our understanding and management of frailty in surgical contexts.

## Figures and Tables

**Table 1:** Demographic data of the patient cohort stratified by frailty.

Variables	Robust	Prefrail/Frail	*p* Value
No. of samples	97	56	
Age (yr.)	61.2 ± 10.5	67.5 ± 8.7	<0.01 *
Gender (female)	90 (92%)	61 (92%)	0.97
BMI (kg/m^2^)	21.8 ± 3.2	22.2 ± 3.9	0.47
BMD (T-score)	−1.0 ± 0.8	−1.3 ± 0.9	0.03 *
Frailty (mFI-11)	0	0.16 ± 0.09	<0.01 *
Schwab-SRS type (T:D:L:N)	8:27:43:19 (8%:28%:44%:20%)	2:9:21:24 (4%:16%:38%:43%)	0.01 *
Level fused	9.8 ± 3.0	9.9 ± 2.8	0.89
LIV (pelvis)	48 (49%)	34 (61%)	0.18
Application of LIF	29 (30%)	20 (36%)	0.46
Application of PSO	7 (7%)	7 (13%)	0.27
Revision surgery	8 (8%)	5 (9%)	0.88
TOS (min.)	244.3 ± 85.2	283.3 ± 115.2	0.02 *
EBL (L)	0.7 ± 0.6	0.6 ± 0.4	0.39
Major complication	40 (41%)	38 (68%)	<0.01

Means and standard deviations. Percentage in brackets. * indicates statistically significant.

**Table 2:** Comparisons of baseline spinal alignment and HRQOLs in ASD patients stratified by frailty.

	Robust	Prefrail/Frail	*p* Value
Spinal alignment			
Cobb angle (°)	56.2 ± 19.1	36.6 ± 9.1	0.13
C7SVA (mm)	57.7 ± 51.3	100.3 ± 66.8	<0.01 *
PT (°)	31.2 ± 13.1	36.4 ± 9.9	0.02 *
PI-LL (°)	34.2 ± 20.4	43.2 ± 23.4	0.04 *
SRS22r score			
Function	3.0 ± 0.8	2.8 ± 0.9	0.11
Pain	3.1 ± 0.8	2.9 ± 0.8	0.10
Self-image	2.1 ± 0.8	2.2 ± 0.7	0.89
Mental heath	2.8 ± 0.9	2.8 ± 1.1	0.82
Satisfaction	3.8 ± 1.2	3.5 ± 1.0	0.32
Total	2.8 ± 0.7	2.7 ± 0.7	0.44

Means and standard deviations; * indicates statistically significant.

**Table 3:** Comparisons of 2 yr post-operative spinal alignment and HRQOLs in ASD patients stratified by frailty.

	Robust	Prefrail/Frail	*p* Value
Spinal alignment			
Cobb angle (°)	18.1 ± 14.3	15.3 ± 10.6	0.41
C7SVA (mm)	43.4 ± 51.0	77.1 ± 66.8	<0.01 *
PT (°)	27.2 ± 10.2	29.3 ± 10.2	0.07
PI-LL (°)	17.4 ± 16.2	20.2 ± 12.7	0.20
SRS22r score			
Function	3.8 ± 0.8	3.5 ± 0.8	0.10
Pain	3.8 ± 0.9	3.6 ± 0.9	0.19
Self-image	3.4 ± 1.0	3.7 ± 0.9	0.32
Mental heath	3.7 ± 0.9	3.7 ± 0.9	0.60
Satisfaction	3.9 ± 0.9	3.8 ± 1.0	0.48
Total	3.7 ± 0.7	3.5 ± 0.7	0.19

Means and standard deviations; * indicates statistically significant.

**Table 4:** Demographic data of the propensity-score-matched patient cohort stratified by frailty.

Variables	Robust	Prefrail/Frail	*p* Value
No. of samples	39	39	
Age (yr.)	62.6 ± 11.1	66.8 ± 9.5	0.25
Gender (female)	38 (97%)	35 (90%)	0.36
BMI (kg/m^2^)	21.4 ± 2.9	22.0 ± 3.5	0.87
BMD (T-score)	−1.0 ± 0.8	−1.4 ± 0.9	0.08
Frailty (mFI-11)	0	0.12 ± 0.09	<0.01 *
Schwab-SRS type (T:D:L:N)	0:9:16:14 (0%:23%:41%:36%)	1:5:19:14 (3%:13%:49%:36%)	0.53
Level fused	9.8 ± 3.6	9.9 ± 2.2	0.89
PSO	2 (5%)	4 (19%)	0.68
LLIF	12 (31%)	15 (39%)	0.64
LIV (pelvis)	17 (44%)	22 (56%)	0.37
TOS (min.)	260 ± 68	275 ± 64	0.50
EBL (L)	0.8 ± 0.8	0.6 ± 0.3	0.20
Baseline spinal alignment			
Cobb angle (°)	41 ±13	39 ± 11	0.79
C7SVA (mm)	84 ± 73	75 ± 55	0.68
PT (°)	33 ± 13	34 ± 9	0.74
PI-LL (°)	37 ± 21	41 ± 20	0.81

Means and standard deviations. Percentage in brackets. * indicates statistically significant.

**Table 5:** Comparison of baseline biomarkers between ASD patients who developed severe adverse events during follow-up and those who did not in prefrail/frail patients.

Biomarkers	SAE−	SAE+	*p* Value
WBC (10^3^/uL)	5.3 ± 1.6	5.3 ± 1.8	0.94
Hb (g/dL)	12.6 ± 1.2	12.0 ± 1.3	0.52
PLT (10^4^/uL)	22.8 ± 7.2	24.4 ± 10.2	0.49
TLC (10^2^/uL)	29.9 ± 8.8	30.0 ± 8.8	0.96
TP (g/dL)	6.7 ± 0.4	6.7 ± 0.6	0.84
ALB (g/dL)	4.0 ± 0.2	3.6 ± 0.6	0.12
BUN (mg/dL)	16.5 ± 4.4	16.4 ± 4.5	0.96
Cr (mg/dL)	0.67 ± 0.18	0.78 ± 0.23	0.05 *
CRP (mg/dL)	0.06 ± 0.12	0.07 ± 0.08	0.86
AST (IU/L)	22.3 ± 8.6	23.5 ± 4.8	0.83
ALT (IU/L)	17.5 ± 9.2	17.5 ± 6.9	0.99
PT-INR (%)	1.0 ± 0.1	0.9 ± 0.1	0.58
aPTT (s)	27.6 ± 3.2	27.4 ± 3.2	0.89

Means and standard deviations. * indicates statistically significant. TLC: Total lymphocyte count.

## Data Availability

The datasets generated and analyzed during the current study are not publicly available but are available from the corresponding author on reasonable request.

## Supplementary Materials

The following supporting information can be downloaded at: https://www.mdpi.com/article/10.3390/jcm13082294/s1, Table S1: Comparison of baseline biomarkers between ASD patients who developed severe adverse events during follow-up and those who did not in the entire population.
