# Supplementary material for: Dysregulation of Inflammatory Pathways in Adult Spinal Deformity Patients with Frailty
Source: J Clin Med. 2024 Apr 16;13(8):2294. doi: 10.3390/jcm13082294 (PMC11051152; doi:10.3390/jcm13082294)
Supplement: Supplementary file 1 [file jcm-13-02294-s001.zip › jcm-2960073-supplementary.pdf]

Journal of
Clinical Medicine

MDPI

**Supplementary Table S1.** Comparison of baseline biomarkers between ASD patients who developed severe adverse events during follow-up and those who did not in the entire population.

| Biomarkers | SAE− | SAE+ | *p* Value |
|---|---|---|---|
| **WBC ($10^3$/uL)** | 5.1 ± 1.7 | 4.9 ± 1.7 | 0.56 |
| **Hb (g/dL)** | 12.4 ± 1.2 | 12.3 ± 1.3 | 0.84 |
| **PLT ($10^4$/uL)** | 24.1 ± 7.1 | 24.2 ± 9.6 | 0.94 |
| **TLC ($10^2$/uL)** | 30.3 ± 9.0 | 30.9 ± 8.2 | 0.79 |
| **TP (g/dL)** | 6.7 ± 0.5 | 6.7 ± 0.6 | 0.90 |
| **ALB (g/dL)** | 4.0 ± 0.3 | 3.9 ± 0.5 | 0.22 |
| **BUN (mg/dL)** | 15.1 ± 4.2 | 16.4 ± 4.7 | 0.11 |
| **Cr (mg/dL)** | 0.67 ± 0.1 | 0.78 ± 0.23 | 0.01 * |
| **CRP (mg/dL)** | 0.23 ± 1.25 | 0.06 ± 0.07 | 0.44 |
| **AST(IU/L)** | 22.3 ± 11.3 | 22.6 ± 4.6 | 0.85 |
| **ALT(IU/L)** | 16.2 ± 7.1 | 16.7 ± 6.0 | 0.66 |
| **PT-INR (%)** | 1.0 ± 0.1 | 0.9 ± 0.0 | 0.54 |
| **aPTT (sec.)** | 28.3 ± 3.4 | 27.0 ± 3.1 | 0.13 |

Means and standard deviations; * indicates statistically significant. TLC: Total lymphocyte count.
